# Interactions between polyphenolic antioxidants quercetin and naringenin dictate the distinctive redox-related chemical and biological behaviour of their mixtures

**DOI:** 10.1038/s41598-021-89314-0

**Published:** 2021-06-10

**Authors:** Monika Baranowska, Zuzanna Koziara, Klaudia Suliborska, Wojciech Chrzanowski, Michael Wormstone, Jacek Namieśnik, Agnieszka Bartoszek

**Affiliations:** 1 Department of Food Chemistry, Technology and Biotechnology, Faculty of Chemistry, Gdansk University of Technology, Narutowicza 11/12, 80-233 Gdańsk, Poland; 2 Department of Physical Chemistry, Faculty of Chemistry, Gdansk University of Technology, Gdańsk, Poland; 3 School of Biological Sciences, Faculty of Science, University of East Anglia, Norwich, UK

**Keywords:** Chemical biology, Electrochemistry, Cancer prevention, Nutritional supplements

## Abstract

Food synergy concept is suggested to explain observations that isolated antioxidants are less bioactive than real foods containing them. However, mechanisms behind this discrepancy were hardly studied. Here, we demonstrate the profound impact of interactions between two common food flavonoids (individual: aglycones quercetin—Q and naringenin—N− or their glycosides rutin—R and naringin—N+ vs. mixed: QN− and RN+) on their electrochemical properties and redox-related bioactivities. N− and N+ seemed weak antioxidants individually, yet in both chemical and cellular tests (DPPH and CAA, respectively), they increased reducing activity of mixtures synergistically. In-depth measurements (differential pulse voltammetry) pointed to kinetics of oxidation reaction as decisive factor for antioxidant power. In cellular (HT29 cells) tests, the mixtures exhibited properties of a new substance rather than those of components. Pure flavonoids did not influence proliferation; mixtures stimulated cell growth. Individual flavonoids tended to decrease global DNA methylation with growing concentration; this effect was more pronounced for mixtures, but not concentration-dependent. In nutrigenomic studies, expression of gene set affected by QN− differed entirely from common genes modulated by individual components. These results question the current approach of predicting bioactivity of mixtures based on research with isolated antioxidants.

## Introduction

Research carried out over the past two decades on the molecular basis of non-infectious chronic diseases such as atherosclerosis, hypertension, diabetes and especially cancer catching major interest, has revealed that all these diseases share a common risk factor, which is the disruption of redox homeostasis often referred to as *oxidative stress*^1,2^. It arises as a result of an increased endogenous level of reactive oxygen species (ROS) due to the body's antioxidant barrier failing and is believed to promote the development of all these illnesses^2,3^. Thus, the assumption followed that exogenous factors capable of neutralizing ROS, e.g. plant antioxidants, could counteract or slow down the development of chronic diseases and support their treatment. Verification of this hypothesis initiated detailed studies on antioxidants present in foodstuffs that might exhibit preventive potential^4^. Indeed, several studies summarized in the meta-analysis comparing food consumption and diet-related chronic diseases revealed decreased risk in the case of diets rich in fruits and vegetables, whole-grain cereals as well as beverages such as wine, coffee and tea, hence products rich in antioxidant phytochemicals^5^. Not surprisingly, it was presumed that these substances once isolated from their natural sources, purified and then consumed in the form of dietary supplements containing higher doses than those achievable in the diet could become powerful chemopreventive agents. This assumption was confirmed by a large body of evidence coming from studies exploiting various experimental in vitro and in vivo models of chronic diseases, including cancer^6,7^. Disappointingly, it has recently been shown in human studies that antioxidant supplements do not exhibit such promising activities. For instance, two meta-analyses of human cohort and case–control investigations with vitamin E^8^ or micronutrient preparations^9^ concluded that low levels of antioxidants had no effect, while high doses might increase both incidence and mortality of cancer and cardiovascular diseases. However, when supplements were based on real plants, such as a specific blend of concentrated polyphenol-rich foods (pomegranate, green tea, broccoli and turmeric), a significant protective effect in men with prostate cancer was observed^10^.

The promising effects of whole foods in contrast to isolated compounds are in line with the food synergy concept, which is defined as an additive or more than additive influence of the combination of different food ingredients on human health^11^. Our earlier study verified this concept by comparing bioactivities of real foods with their isolated major antioxidant. This showed that the biological effects of extracts of berry fruits vastly differ from those exhibited by anthocyanin cyanidin-3-*O*-glucoside^12^. Some other reports also indicated the importance of the interactions between different bioactive compounds and food matrix components that turned out to be cooperating factors, which determine the final bioactivity of foods^13–17^. Our recent mechanistic investigations involving step-wise reconstitution of cocoa composition of bioactives also supported the idea of food synergy, but demonstrated that the biological effects of samples with complex compositions are not just a combination of the activities displayed by individual components^13^. All these observations suggested that when considering redox related bioactivities of isolated antioxidants versus their mixtures, the interactions between components must be taken into account. The growing complexity of a mixture of phytochemicals seemed to create a new redox-active substance rather than enrich the mixture with new activities characteristic of the compound added, which is inferred by the food synergy concept.

In the current research, we simplified the experimental system by limiting it to only two core structures in order to delve into details of their interactions in the context of chemical structure, redox reactivity and redox-related bioactivities, so to enable better understanding and prediction of the chemopreventive potential of antioxidants. The phytochemicals used for this purpose were common antioxidants present in various herbs, vegetables and fruits, especially in citrus fruits, namely: flavonols represented by quercetin (Q) and its rhamnoside–rutin (R) and flavanones by naringenin (N−) and its neohesperidoside naringin (N+) as well as two mixtures of these compounds (QN−, RN+). The chemical component of the study embraced determinations giving some insight into thermodynamics and kinetics of oxidative processes, i.e.: DPPH test, potentiometric titration and differential pulse voltammetry (DPV). The biological tests examined the impact of the studied samples on cell growth (MTT test), cellular antioxidant activity (CAA assay), genotoxicity (comet assay), global DNA methylation level (epigenetic version of comet assay) and the expression of 84 redox-related genes (real-time PCR array-based technologies). The biological experiments were carried out using the recommended for nutritional studies colon adenocarcinoma HT29 cell line as a model of the intestinal epithelium that may be exposed to relatively high concentrations of ingested antioxidants.

## Results

**Figure 1:**
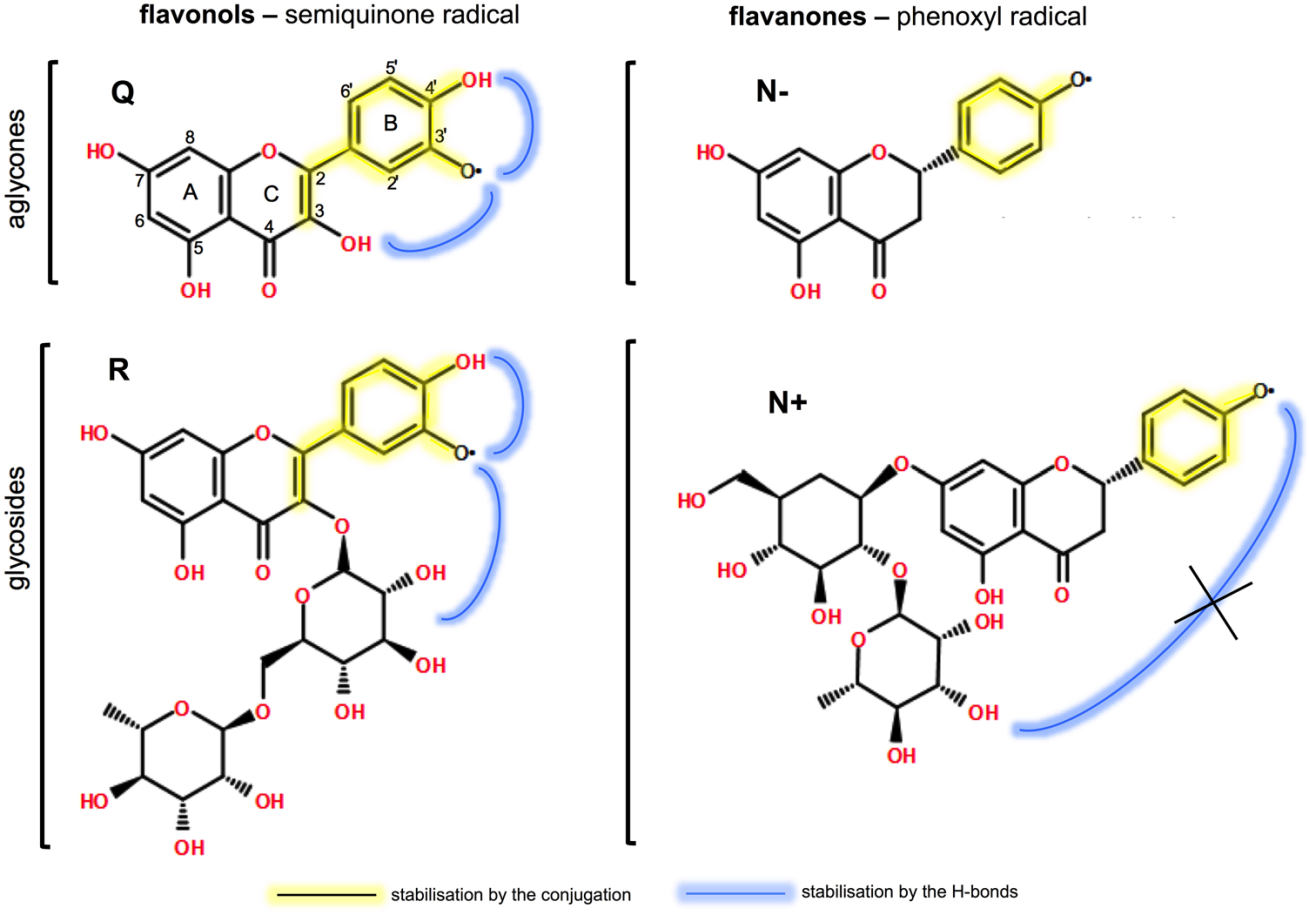
The chemical structures of radicals formed upon the first stage of oxidation of flavonoids under study with indicated redox-active moieties and the possible sites of intramolecular H-bond formation. The hydrogen bonds may be formed directly or via water molecule depending on structural circumstances. The abbreviated names of flavonoids refer to: Q-quercetin, R-rutin, N−-naringenin, N+ -naringin.

Our earlier investigations that compared redox-related properties of cocoa powder and its main constituents pointed to the importance of interactions between polyphenolic components of the mixture on overall antioxidant activity. In the current research, we simplified the experimental system to examine such interactions in more detail for a pair of flavonoids that are common food components. Two flavonoids were chosen, both in the form of aglycones and glycosides. The flavonols were represented by quercetin (Q) and its rhamnoside–rutin (R) and flavanones by naringenin (N−) and its neohesperidoside naringin (N+). These polyphenols differ in the number and location of redox-active hydroxyl groups as well as the ability to form intramolecular H-bonds, i.e., three structural features that may interfere with reducing properties of antioxidant compounds. As shown in Fig. 1, the intermediate semiquinone radicals formed in the first step of oxidation of catechol moiety in ring B of Q or R can be stabilized in two ways^18,19^. The first way is the conjugation of both the core structure over the B and C rings and the second–H-bond formation with vicinal OH group or substituents in C ring. Especially in R, the presence of hydroxyl groups in sugar substituent in position 3 of ring C may further enhance this stabilization effect due to more possibilities of formation of H-bonds (directly or via water molecule). In contrast, the intermediate phenoxyl radical in N+ or N− is stabilized neither by conjugated double bonds involving also ring C nor H-bonding with neighbouring substituents. Moreover, in N+, the sugar moiety is attached to ring A and thus is too far to form H-bond with the radical in ring B. One can expect these structural features to influence the redox activity of studied flavonoids.

### Antioxidant activity by chemical tests

**Figure 2:**
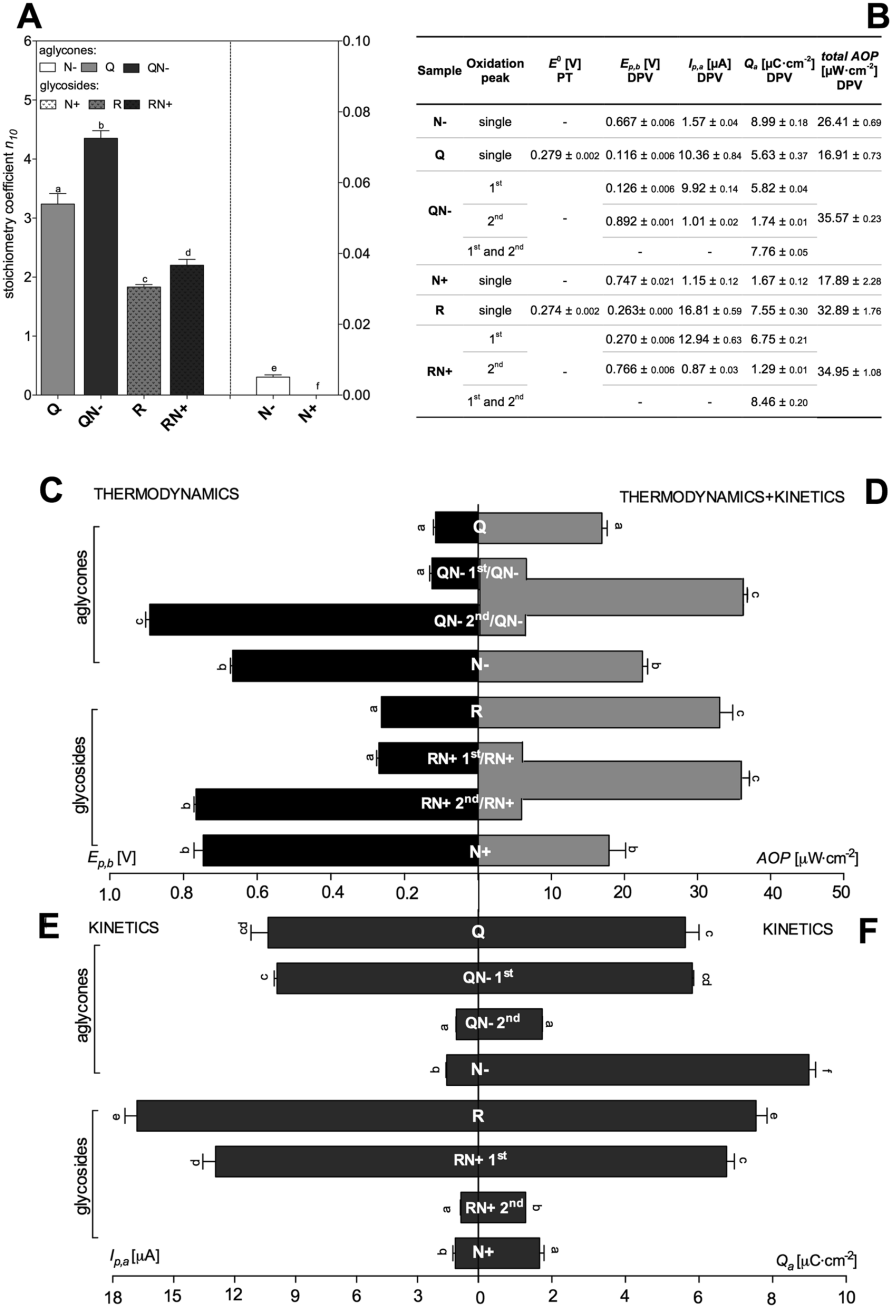
Comparison of antioxidant properties of individual flavonoids (Q, R, N−, N+) and their mixtures (QN−, RN+). (**A**) Antioxidant activity expressed as *stoichiometry coefficient n*_*10*_ calculated based on the DPPH test (as defined in section “Antioxidant activity by differential pulse voltammetry”). (**B**) The values of standard reduction potential (*E*^0^) measured by potentiometric titration (PT) and potential of oxidation peak *vs* standard hydrogen electrode (*E*_*p,b*_), anodic peak potential (*I*_*p,a*_), charge density of the process (*Q*_*a*_), and the antioxidant power considering 1^st^ and 2^nd^ stage of oxidation process (*total AOP*) at concentration 3 mM determined by DPV (*v* = 0.1 V·s^−1^). (**C**) Thermodynamic characterization by *E*_*p,b*_. (**E**,**F**) kinetic properties described by *Q*_*a*_ and *I*_*p,a*_. (**D**) combination of thermodynamic and kinetic parameter expressed as *antioxidant power* (*AOP*). In the case of mixtures, two anodic peaks were observed (1^st^ related to Q/R and 2^nd^ related to N−/N+ in the mixture). All results are given as means ± SD of three independent determinations. Different letters indicate a significant difference determined by one-way ANOVA with Tukey’s test (*p* ≤ 0.05).

The determination of reducing properties for the studied polyphenols and their mixtures was performed by two chemical assays at 37 °C to match the cellular conditions of redox processes. The first method was the commonly used batch spectrophotometric DPPH test; the results for individual flavonoids and their mixtures are presented in Fig. 2A. They are expressed as stoichiometry values *n*_*10*_, where the number 10 refers to the duration of the reaction—10 min. By introducing the time parameter into measurements, a kinetic aspect became incorporated into antioxidant activity assessment as has been described earlier^13^. In these determinations, both aglycones displayed stronger reducing properties than corresponding glycosides as had also been formerly shown with this test^20,21^, while flavonols were more active than flavanones. Q was the most efficient compound in scavenging DPPH^·^ radical and was followed by R. Despite negligible reactivity towards DPPH^·^, both flavanones, including N+ that by itself exhibited no redox properties within the 10 min period of the reaction, significantly increased the total antioxidant activity of the mixtures, in the case of both aglycones QN− and glycosides RN+.

The second method involved potentiometric titration (PT) that allows measurement of standard reduction potential (*E*^0^), and thus evaluated the thermodynamic ability of pure compounds to gain electrons. The determined values of *E*^0^ confirmed that Q and R are strong reducing compounds (Fig. 2B). However in PT, R accepted donor electrons more willingly than Q. The determination of *E*^0^ for N− and N+ was not possible due to very slow electron transfer during the oxidation process (slower for N+). PT measures the difference in potential between the reference electrode and the measuring electrode after adding each portion of the titrant. The steady potential means that the quotient of reaction (*Q*) between titrant and analyte is stable (*Q* = *constant*). If the rate of charge transfer during a reaction is low (low currents in voltammetry), then it takes a long time to stabilize the *Q* in PT. Consequently, for very slow reactions, the potentiometric titration curve is difficult to obtain and thus, the found value of *E*^0^ is less reliable.

### Antioxidant activity by differential pulse voltammetry

The chemical tests used suggested that elucidation of antioxidant action of polyphenols must take into consideration kinetic aspects, where the stability of intermediate radicals could play a role. As illustrated in Fig. 1, semiquinone radicals formed upon the first stage of flavanol oxidation are much better stabilised than phenoxyl radicals arising upon flavanone oxidation. This relation is illustrated in Fig. 1 and may affect the rate of redox processes. Therefore, the reduction–oxidation properties of studied pure antioxidants and their mixtures were further analysed with the aid of differential pulse voltammetry (DPV). Since this technique enables monitoring of both thermodynamic and kinetic aspects of oxidation reactions, both finally combined in a parameter called antioxidant power (*AOP*)^13^.

The observations made with DPV measurements (Fig. 2B–F) contradicted those acquired with the DPPH test (Fig. 2A). Surprisingly, DPV revealed that Q described in the literature as an excellent reductant, when considering thermodynamic aspects only (anodic peak potential, *E*_*p,a*_), proved the weakest antioxidant (Fig. 2B,C). Thermodynamically, R was a slightly stronger antioxidant. Interestingly, N− and N+ that are considered in the literature as weak antioxidants, exhibited thermodynamically the highest values of *E*_*p,b*_, meaning that they were very strong reducing agents. For both flavonoid classes, glycoside moiety increased antioxidant activity of aglycones. However, kinetics-related parameters (Fig. 2E,F), i.e., anodic current (*I*_*p,a*_) and charge density (*Q*_*a*_), revealed that oxidation of N+ is the slower process compared to oxidation of Q and R. Similarly, anodic current (*I*_*p,a*_) was lower for N− than for Q and R, but the charge transfer for this compound reached the highest value.

In the case of mixtures, two anodic peaks (1^st^ and 2^nd^) on voltammetric curves were detected as could be expected for two-component mixture. The determined values of anodic peak potentials (*E*_*p,a*_) indicated that 1^st^ peak observed reflects oxidation of flavonols, while 2^nd^ peak the oxidation of flavanones (Supplementary Materials—Fig. S1). In most cases, the presence of the other component in a mixture influenced the thermodynamics and/or kinetics of the redox process compared to oxidation of the pure compounds. For example, for QN−, the value of *E*_*p,b*_ for 1^st^ peak of oxidation was equal to anodic peak potential of Q oxidation. However, the 2^nd^ anodic peak corresponding to N− oxidation and the potential of this transition was higher than the anodic potential of pure N− (Fig. 2C). The opposite situation was observed for kinetics of this reaction. The *I*_*p,a*_ and *Q*_*a*_ of 1^st^ anodic peak of QN− were close to kinetic parameters of pure components’ oxidation (Fig. 2E,F), while the charge exchanged during 2^nd^ step of QN− oxidation was much lower than that for N− oxidation (Fig. 2F). These combined thermodynamic and kinetic effects resulted in the enhancement of *AOP* (Fig. 2D) of this mixture, which is in accord with the results of DPPH test.

### Cytotoxicity assessment

**Figure 3:**
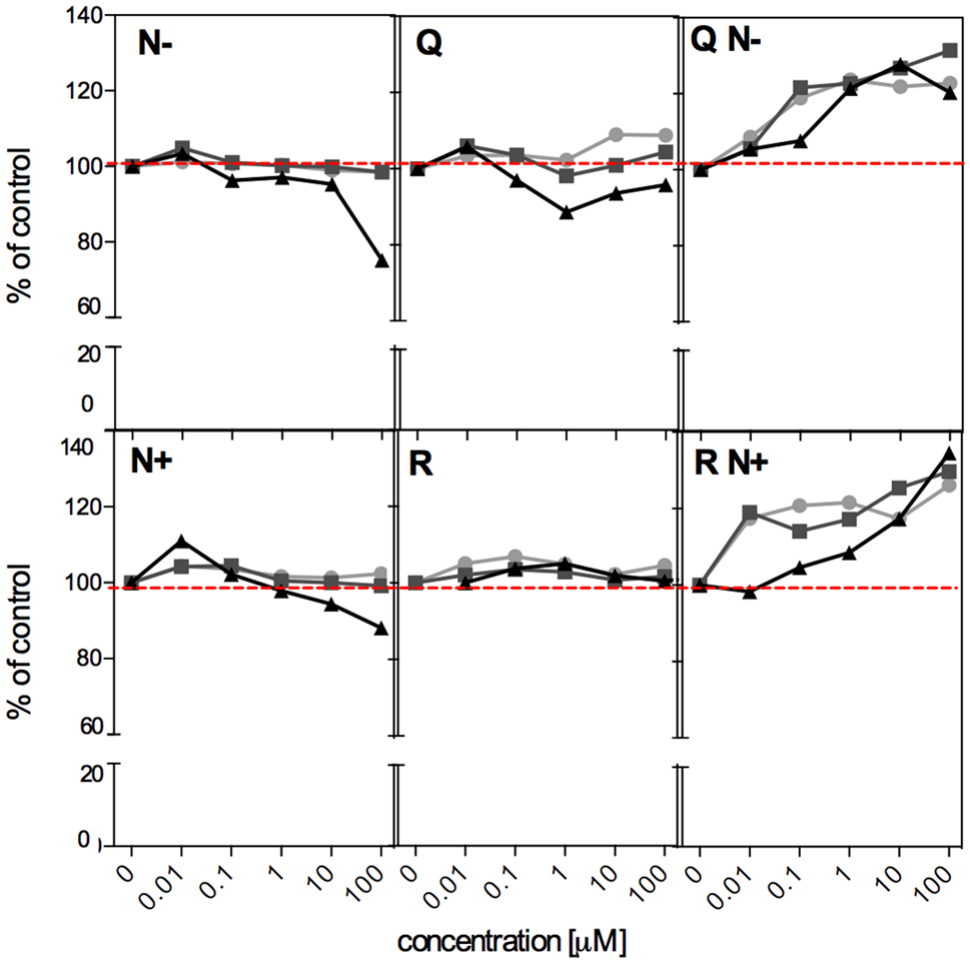
Inhibition of growth of HT29 cells determined by MTT test after 6 (circles), 24 (squares) and 72 h (triangles) exposure to individual flavonoids (0.01–100 μM) and their mixtures containing equal concentrations of each compound (0.01–100 μM). Results represent means of three independent experiments carried out in triplicates (for clarity SD values are not presented, but in all cases were lower than 13%).

The impact of the studied flavonoid aglycones (Q, N−), glycosides (R, N+) and their mixtures (QN−, RN+) on intestinal cell growth was assessed by MTT test. The human colon adenocarcinoma HT29 cell line was chosen as a model of alimentary tract epithelium, *i.e.* the tissue in direct contact with ingested food ingredients such as polyphenols. The cells were treated with individual flavonoids and their mixtures at physiological concentrations potentially occurring in the blood (0.01–1 μM)^22–24^ or concentrations reachable in the alimentary tract (10–100 μM) after food ingestion^25–27^. The dose response curves for 6, 24, and 72 h treatments are presented in Fig. 3.

Individual compounds did not significantly influence the cell growth at any of the investigated concentrations, for neither short nor prolonged treatments. The exception was the highest concentration of N− that after 72 h inhibited cell growth down to 75% compared to control. In contrast, the investigated mixtures (QN−, RN+) significantly stimulated cell growth in a concentration-dependent manner for all exposure times tested. This effect was observed at low concentrations (0.01–1 μM) being reachable in the bloodstream and was even more potent at higher concentrations (10–100 μM) to which epithelial cells of the alimentary tract may be exposed. Only in the case of the highest concentration of QN−, after 72 h treatment, the stimulation ceased, probably due to inhibitory effects observed under such conditions for N−.

### Cellular antioxidant activity

The efficiency of purified flavonoids and their mixtures in supporting the endogenous antioxidant barrier of HT29 cells was verified with the aid of CAA assay. This method relies on the ability of a sample containing redox-active compounds to inhibit or promote the oxidation of the probe absorbed by cells to its fluorescent form. The attenuation of the probe oxidation, observed as the quenching of fluorescence, is a measure of the reducing capacity of antioxidants in the cells (positive CAA values), while the increase of probe oxidation denotes their prooxidative activity (negative CAA values)^28^. The determinations were carried out for aglycones and glycosides at concentrations reflecting both physiological—endogenous—and food derived—exogenous—gut exposures. The incubation with studied flavonoids was carried out for standard recommended period of 1 h^28^ for aglycones and glycosides. The prolonged treatments (3 and 6 h) aimed at monitoring of the kinetics of redox response in the cellular model applied were used only in the case of aglycones, because of their more prominent impact on cellular antioxidant activity.

**Figure 4:**
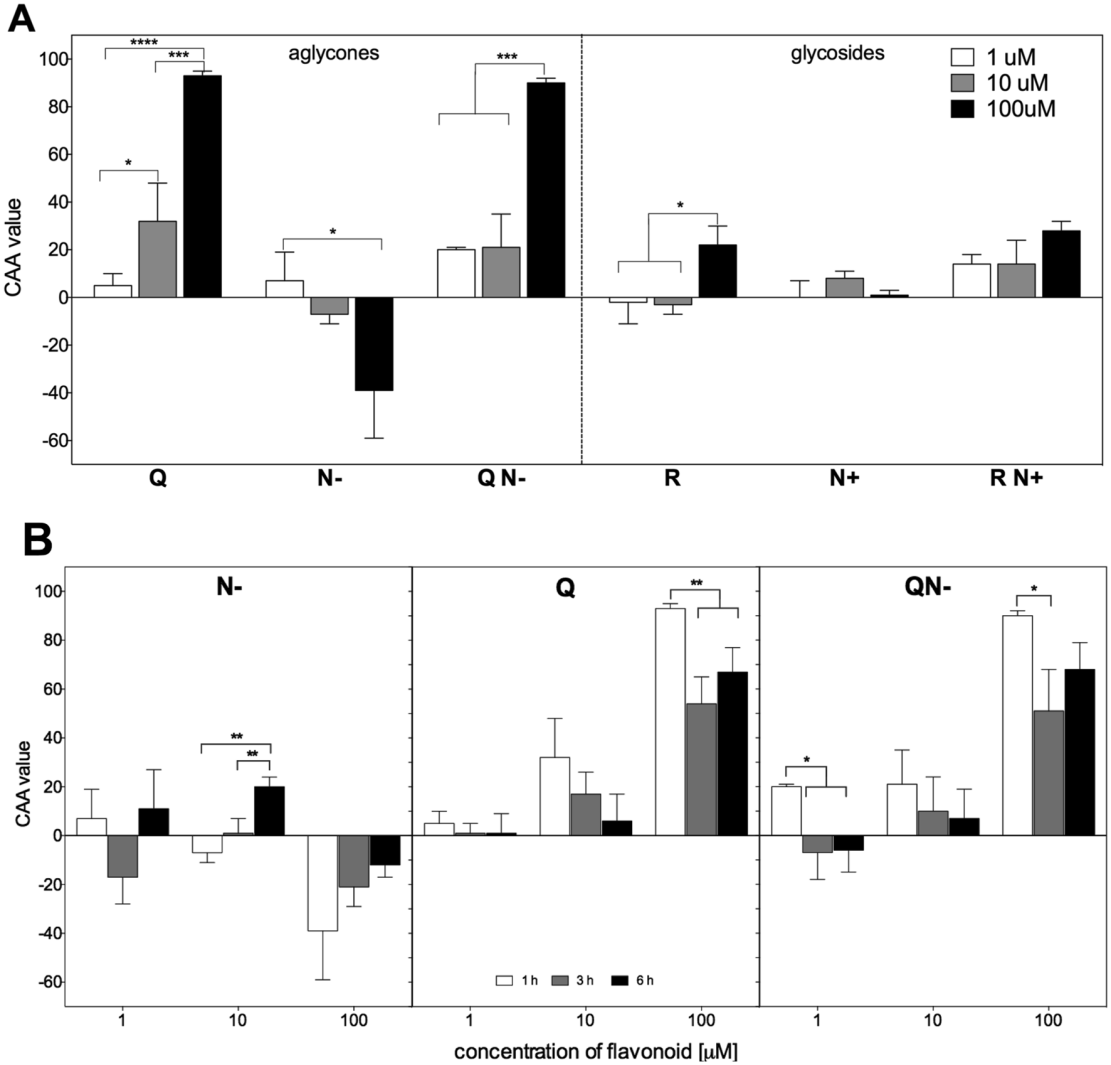
(**A**) The concentration dependence of cellular antioxidant activity of individual aglycones and glycosides (1–100 μM) and their mixtures containing equal concentrations of each compound (1–100 μM) after 1 h treatment. (**B**) The kinetics of changes in cellular antioxidant activity determined for Q and N− (1–100 μM) and their mixture QN− containing equal concentrations of each compound (1–100 μM) after 1, 3 and 6 h treatment. Results are means ± SD of three independent experiments carried out in triplicates. Significantly different values determined by one-way ANOVA with Tukey’s test are marked as: *p ≤ 0.05, **p ≤ 0.01, ***p ≤ 0.001, ****p ≤ 0.0001.

The investigated flavanones and flavonols differed in their impact on redox status of HT29 cells. In the case of individual aglycones, the defined concentration dependent responses were observed after 1 h exposure. However, flavonol—Q antioxidant activity increased with concentration applied, while in the case of flavanone—N− the gradual enhancement of the pro-oxidative effect was observed (Fig. 4A). The dose dependency of individual glycosides was less evident; only R at its highest concentration convincingly increased the cellular antioxidant activity (Fig. 4A). Interestingly, both mixtures displayed enhanced antioxidant activity, apparently not influenced by the pro-oxidative effect seen for individual compounds.

Figure 4B presents the kinetics of changes of CAA values determined after 1, 3 and 6 h treatment of HT29 cells with aglycones. For the lowest concentration (1 μM), matching physiological exposures, the time dependence was not observed neither for individual aglycones nor their mixture. However, the influences of higher concentration on CAA values were clearly time-dependent. The prolonged exposures decreased both the pro-oxidative effect of N- as well as the antioxidant activity of Q and QN−.

### Genotoxic effects

**Figure 5:**
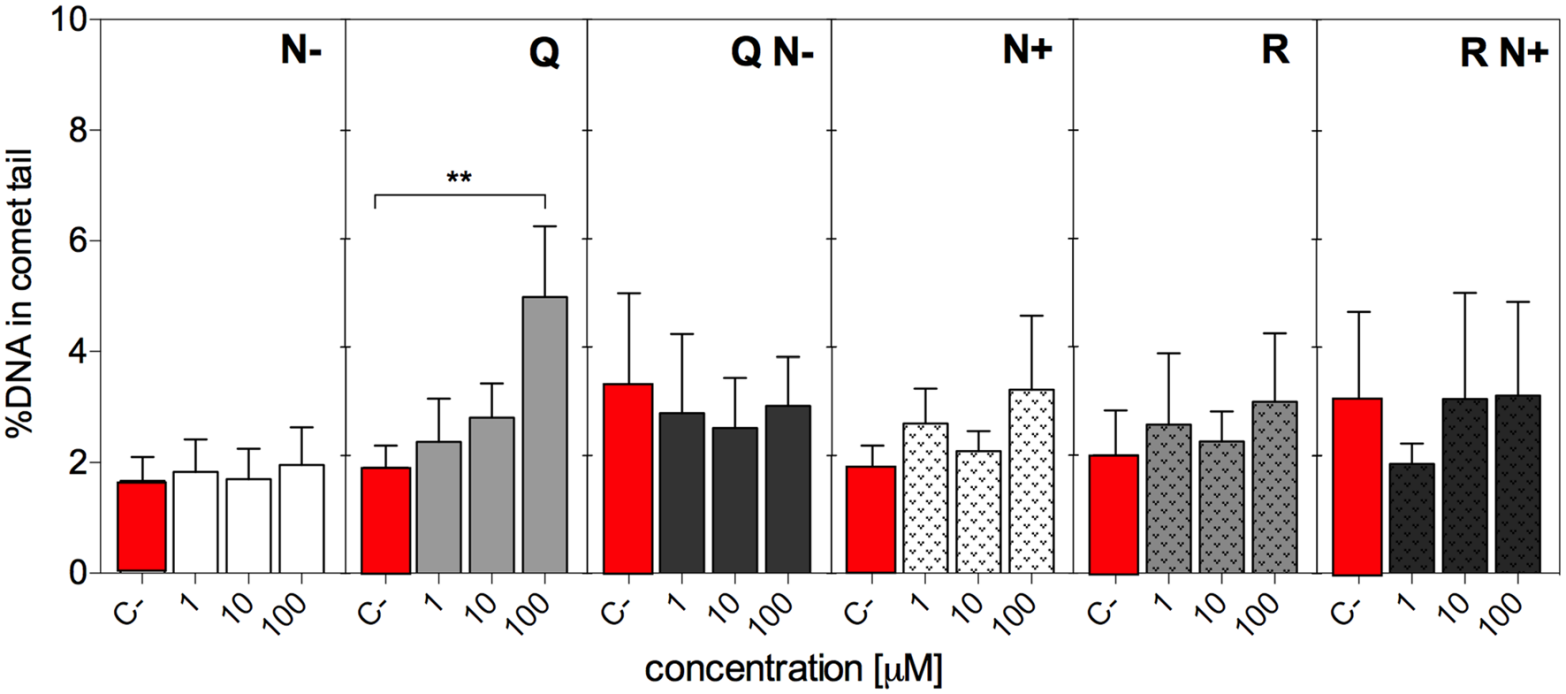
Genotoxicity of tested flavonoids (1–100 μM) and their mixtures containing equal concentrations of each compound (1–100 μM) in HT29 cells evaluated with the aid of comet assay and expressed as %DNA in the comet tail. Results represent means ± SD of three independent experiments. Negative control (C-) refers to cells treated with solvent only. Significantly different values determined by one-way ANOVA with Dunnett's test are marked as: **p ≤ 0.01.

The impact of individual flavonoids or their mixtures on DNA integrity was assessed by comet assay, a useful method for detecting DNA strand breaks in single cells. Under the treatment conditions, none of the investigated flavanones (N−, N+) was genotoxic per se to HT29 cells, regardless of the presence of glycoside moiety and applied concentration (Fig. 5). Even at the highest 100 μM concentration, N− did not promote DNA fragmentation. In the case of flavonols, only the tested aglycone—Q—increased DNA damage at 100 μM concentration. However, this effect was not observed for mixture QN− containing 100 μM of each component. The glycoside of quercetin—R—and mixture RN+ did not impact the integrity of DNA (Fig. 5).

### Global DNA methylation

DNA methylation is a reversible epigenetic modification and impairments of methylome profile occur at the early stage of carcinogenesis; therefore, it has become a promising target for preventive strategies. Since DNA methylation is the cellular process that is influenced by cellular redox status, we tested if this epigenetic modification might also be affected by the studied redox-active compounds. To assess the modulation of global DNA methylation by tested antioxidant flavonoids and their mixtures, the modified version of comet assay was applied. The sensitivity and specificity of such a modified assay are greatly enhanced owing to the use of restriction endonucleases that are sensitive to methylation of restriction sequences. Here, to measure the global DNA methylation level in single cells by comet assay, the isoschizomeric properties of two restriction endonucleases MspI and HpaII were exploited. These enzymes recognize the same sequence (5′-CCGG-3′), but show different sensitivity toward methylated cytosine. HpaII cleaves only non-methylated sequences, while MspI is methylation insensitive and cuts both non-methylated and fully methylated restriction sites.

**Figure 6:**
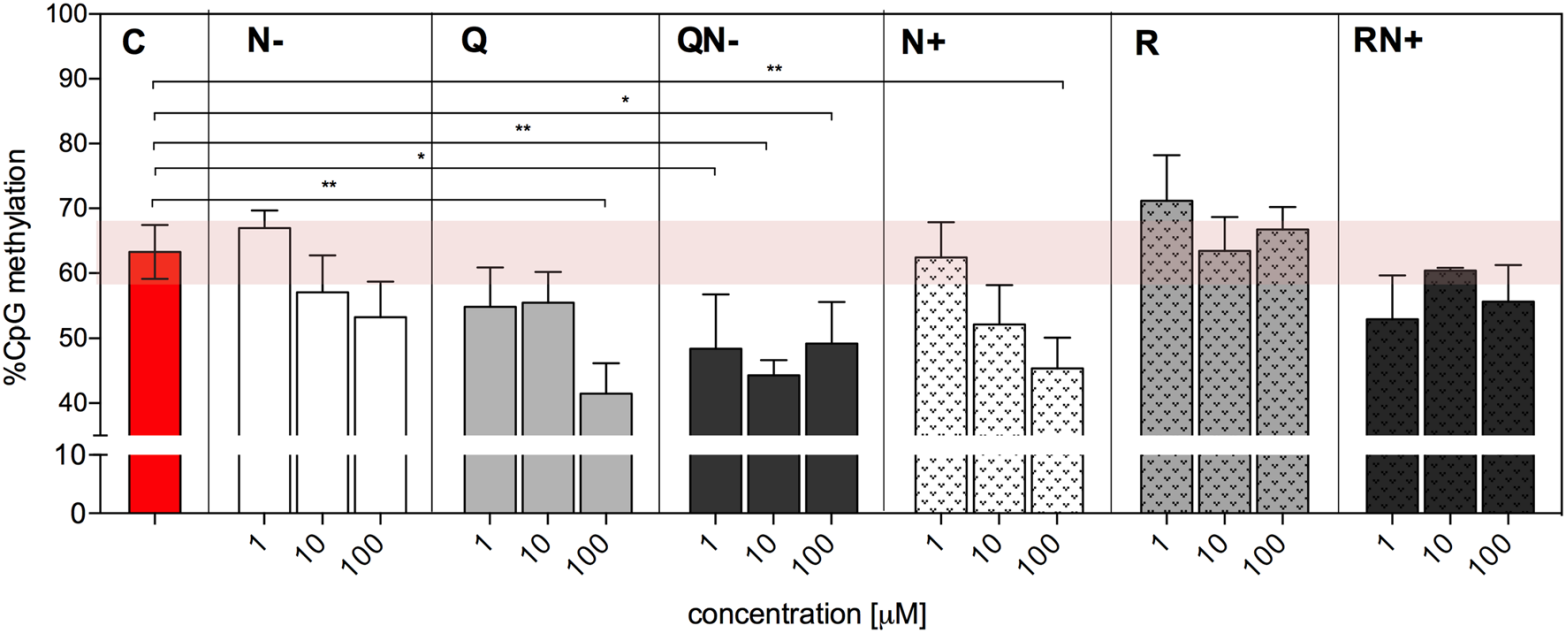
Global DNA methylation of HT29 cells exposed to flavonoids (1–100 μM) and their mixtures containing equal concentrations of each compound (1–100 μM) determined by the epigenetic version of the comet assay. The percentage of DNA in the tail in the case of MspI treatment represents all CCGG sites in cellular DNA, while HpaII is presumed to digest only non-methylated sequences. The global DNA methylation was calculated as described under Materials and Methods section. Control (C) refers to cells treated with solvent only. Results represent the means of three independent experiments. Significantly different values determined by one-way ANOVA with Dunnett's test are marked as: *p ≤ 0.05, **p ≤ 0.01.

Both investigated individual aglycones tended to diminish global DNA methylation of HT29 cells with growing concentration compared to control cells. For the highest concentration of Q and all treatments with aglycone mixture QN−, this decrease reached statistical significance (Fig. 6). A similar trend was observed for pure flavanone glycoside N+, but not for flavanol glycoside R. In contrast to QN−, RN+ did not exhibit a stronger ability to lower DNA methylation than their components individually.

### Microarray analysis

**Figure 7:**
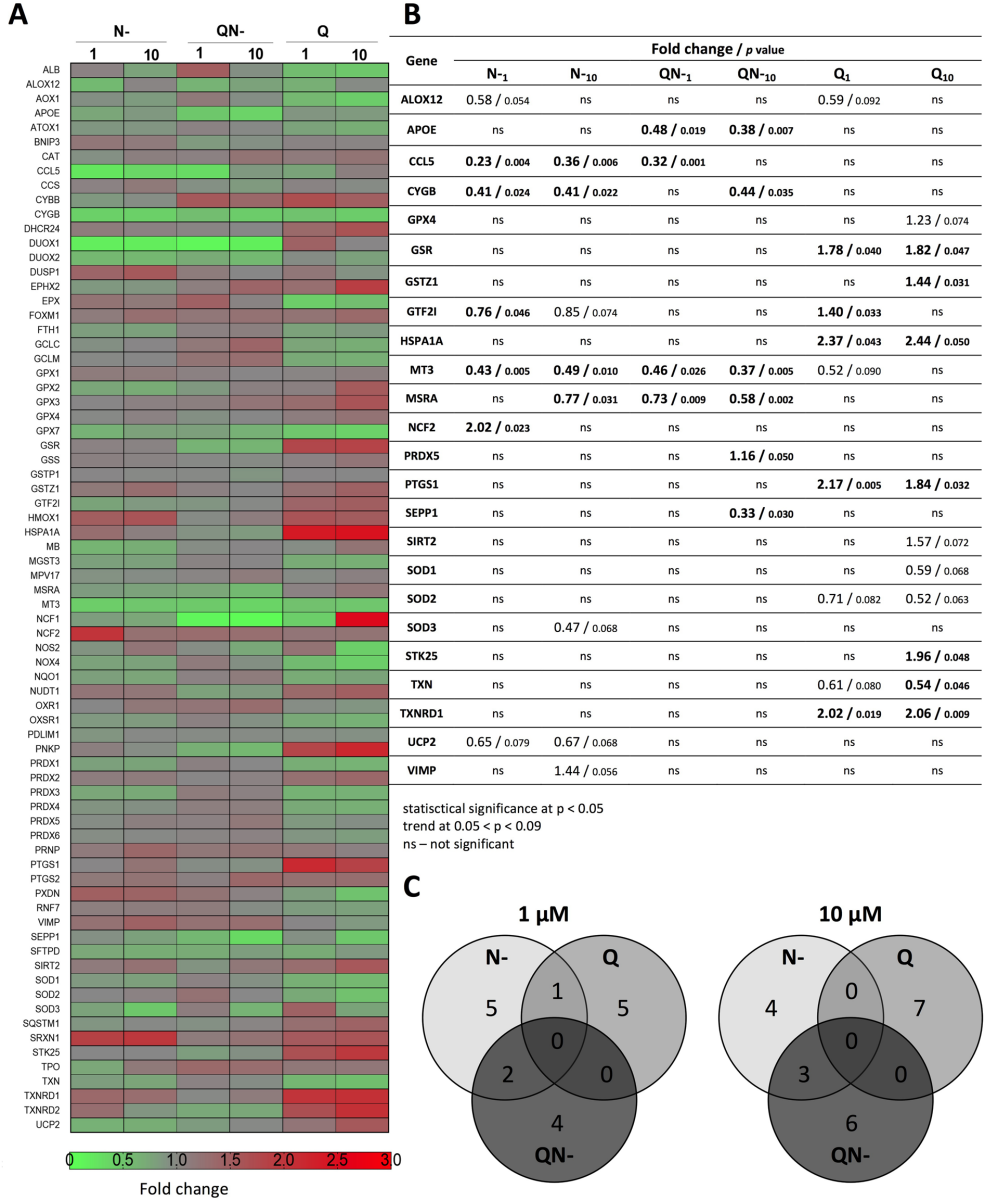
(**A**) heat map presenting the modulation of oxidative stress response and expression of antioxidant defence genes in HT29 cells after 24 h treatment with Q, N− at concentrations 1 or 10 μM and their mixture containing equal concentrations of each compound (1–10 μM). (**B**) Fold changes in the expression of genes as a result of treatment and probability values evaluated by the unpaired Student’s t-test. Statistically significant changes in the expression of genes are highlighted in boldface. (**C**) Venn diagram showing common genes regulated by Q, N− and QN− at *p* < 0.05. The results are calculated based on three independent experiments.

The present work aimed to compare the relative impact of the studied aglycones (Q, N−) and their mixture (QN−) on expression of redox related genes. The set of 84 genes (details in Table S1 in Supplementary Materials) embraced genes encoding proteins relevant for antioxidant activity, superoxide release and metabolism, the activity of peroxidases and oxidoreductases, as well as those essential for inflammation, apoptosis, regulation of cell cycle, and other processes associated with oxidative stress. Figure 7A shows a heat map illustrating the modulation of gene expression within the array investigated, while Fig. 7B shows the fold changes for selected genes determined for HT29 cells in response to 24 h treatment with individual compounds (Q, N−) and their mixture (QN−) at two concentrations: physiological (1 μM) and dietary relevant (10 μM). Venn diagram (Fig. 7C) summarises the genomic analysis by indicating common genes with expression levels that were significantly changed (*p* < 0.05) by the treatment.

The investigated aglycones displayed different nutrigenomic activity, additionally modified by the concentration applied to cells. Flavanone N− at 1 μM significantly decreased expression of *CCL5*, *CYGB*, *GTF2I*, *MT3* (*p* < 0.05) as well as showing some tendency to down-regulate *ALOX12* and *UCP2* transcription (0.05 < *p* < 0.09). The increased expression caused by N− was observed for *NCF2* gene only (*p* < 0.05). These genes, though in one or another way, related to cellular redox status, do not fall into any specific common pathway nor are involved in any coordinated process. The protein encoded by *CCL5* belongs to a group of inflammation-relevant genes, while the cytoglobin gene (*CYGB*) functions as a tumour suppressor gene^29,30^. GTF2I protein acts as a general transcription factor and is involved in the coordination of cell growth and division^31^. So, the other gene down-regulated by N− at 1 μM gene—*MT3*—may cooperate with it, because although it plays a role in zinc and copper homeostasis, it is also known as growth inhibition factor^32^. The enzyme encoded by *ALOX12* acts on different polyunsaturated fatty acid substrates to generate bioactive lipid mediators^33^. The protein coded by *UCP2* has been described as a mitochondrial scavenger of ROS^34^. The only up-regulated gene by N− at 1 μM was *NCF2* that encodes a cytosolic protein required for the activation of the NADPH oxidase system responsible for superoxide production^35^.

This flavanone applied to HT29 cells at 10 μM influenced the expression of 5 genes, which were also down-regulated by its lower dose, namely: *CCL5*, *CYGB*, *MT3* (*p* < 0.05) as well as *GTF2I* and *UCP2* (0.05 < *p* < 0.09). Additionally, in contrast to lower dose, N− at 10 μM showed tendency to decrease expression of *SOD3* (0.05 < *p* < 0.09). The latter gene codes for a protein with superoxide dismutase activity, i.e., the antioxidant enzyme catalysing the dismutation of superoxide radicals to hydrogen peroxide and oxygen^36^. The slight increase in expression caused by N− at 10 μM was only observed for *VIMP* gene (0.05 < *p* < 0.09) that is involved in the degradation process of misfolded endoplasmic reticulum (ER) luminal proteins^37^.

Q at concentration amounting to 1 μM also tended to decrease expression of *ALOX12* and *MT3* (0.05 < *p* < 0.09) as well as *SOD2* and *TXN* (0.05 < *p* < 0.09). The latter gene codes for protein that belongs to the family of thioredoxins and acts as endogenous antioxidant facilitating the reduction of other proteins^38^. In contrast, to N−, Q at 1 μM up-regulated *GTF2I* gene (*p* < 0.05). The other genes whose expression was significantly elevated by Q at 1 μM were *GSR*, *HSPA1A*, *PTGS1* and *TXNRD1*; all play key roles in building cellular defences against oxidants. Up-regulation of *GSR* is crucial for maintaining redox homeostasis in cells, because the encoded protein maintains high levels of reduced glutathione in the cytosol^39^. In turn, HSPA1A chaperone is needed to correct any occurring misfoldings, also those resulting from exposure to antioxidants. The latter may shift the redox balance towards a reduced state, leading to the more probable reduction of disulfide bridges to sulfhydryl groups and thereby changing the protein structure and thus function^40^. *PTGS1* gene encodes protein that is yet another member of the antioxidant enzyme family, namely prostaglandin synthase-2^41^. The fourth mentioned gene—*TXNRD1*—codes for thioredoxin reductase that keeps thioredoxin (TXN) in the reduced state^42^.

Similarly to 1 μM, Q at higher concentration (10 μM) also decreased expression of *TXN* (*p* < 0.05) and *SOD2* (0.05 < *p* < 0.09). Furthermore, the drop in expression was observed for *SOD1* (0.05 < *p* < 0.09). The investigated flavonol at 10 μM influenced the expression of also 4 genes up-regulated by its lower concentration: *GSR*, *HSPA1A*, *PTGS1* and *TXNRD2* (*p* < 0.05). The additional up-regulated genes by the higher Q concentration included *GSTZ1* and *STK25* (*p* < 0.05) as well as *GPX4* and *SIRT2* (0.05 < *p* < 0.09). Protein encoded by *GSTZ1* is a member of the glutathione S-transferas family that are key enzymes implicated in the detoxification of electrophilic molecules by conjugation with GSH^43^, while *STK25* codes for serine/threonine kinase 25, a protein activated by oxidative stress that induces apoptotic cell death^41^. In turn, *GPX4* codes for glutathione peroxidase 4, which supports the antioxidant barrier of the cell by catalysing the reduction of peroxides by glutathione^44^. The up-regulation of *SIRT2*, encoding NAD-dependent protein deacetylase, which deacetylates internal lysines present in, e.g. histones or transcription factors, plays a role in the modulation of key biological processes, such as cell cycle control, cell differentiation or genomic integrity^45,46^.

The mixture of aglycones at 1 μM decreased expression of *APOE*, *CCL5*, *MT3* and *MSRA* to the extent of reaching statistical significance (*p* < 0.05). The apolipoprtotein encoded by *APOE* is a core component of plasma lipoproteins and is involved in their production, conversion and clearance^47^. The protein encoded by *MSRA* carries out the enzymatic reduction of methionine sulfoxide to methionine, thus this protein functions in the repair of oxidatively damaged proteins to restore biological activity^48^. The mixture QN− at 10 μM, similarly to lower dose, decreased expression of *APOE*, *MT3* and *MSRA* (*p* < 0.05). However at a higher concentration, the set of down-regulated genes was extended to incorporate *CYGB* and *SEPP1* (*p* < 0.05). The latter gene *SEPP1* encodes selenoprotein P, the extracellular glycoprotein that has an antioxidant role and appears to be associated with endothelial cells^49^. The mixture QN− only slightly potentiated the expression of *PRDX5* gene that codes for a member of the peroxiredoxin family of antioxidant enzymes, whose role is to reduce hydrogen peroxide and alkyl hydroperoxides^50^.

## Discussion

The scientific basis of epidemiological observations that indicate whole fruits and vegetables are more efficient in preventing chronic non-infectious diseases than bioactive compounds isolated from them is poorly understood. The food synergy concept, which assumes additive or even synergistic influence of different food ingredients on human health, has been proposed as a possible explanation^11^. Such reasoning was however undermined to some extent by our previous research in which we compared bioactivity of differently pigmented vegetables (brassicas) as well as berry fruits, white *vs* anthocyanins containing, and found no pattern of activity that could be ascribed to coloured varieties, with the exception of higher antioxidant activity in chemical tests^12,51^. Nor were the biological effects matched to those of isolated cyanidin-3-*O*-glucoside investigated at the concentration occurring in studied plant material^12^. Further mechanistic investigations on cocoa reconstitution also showed no additive/synergistic biological effects of mixtures of components as predicted by the food synergy concept, but rather entirely altered bioactivity^13^. The subsequent mixtures of cocoa polyphenols seemed to behave as new substances. We hypothesized that the interactions between individual components in the mixture could create a new entity displaying modified physicochemical properties resulting in novel biological activities. Indeed, the variety of possible interactions between polyphenols (hydrogen, *π*, hydrophobic, chelating, covalent and electrostatic), discovered during investigations on their application as stabilisers of self-assembled nanoparticles, were shown to produce a range of structures differing in functionality^52^. It was also observed that these phytochemicals usually exert more than one type of stabilising attractive forces. In the current research, we investigated how the interplay of often competing interactions in a mixture of just two polyphenolic antioxidants impacted its redox-related activities compared to individual components.

**Figure 8:**
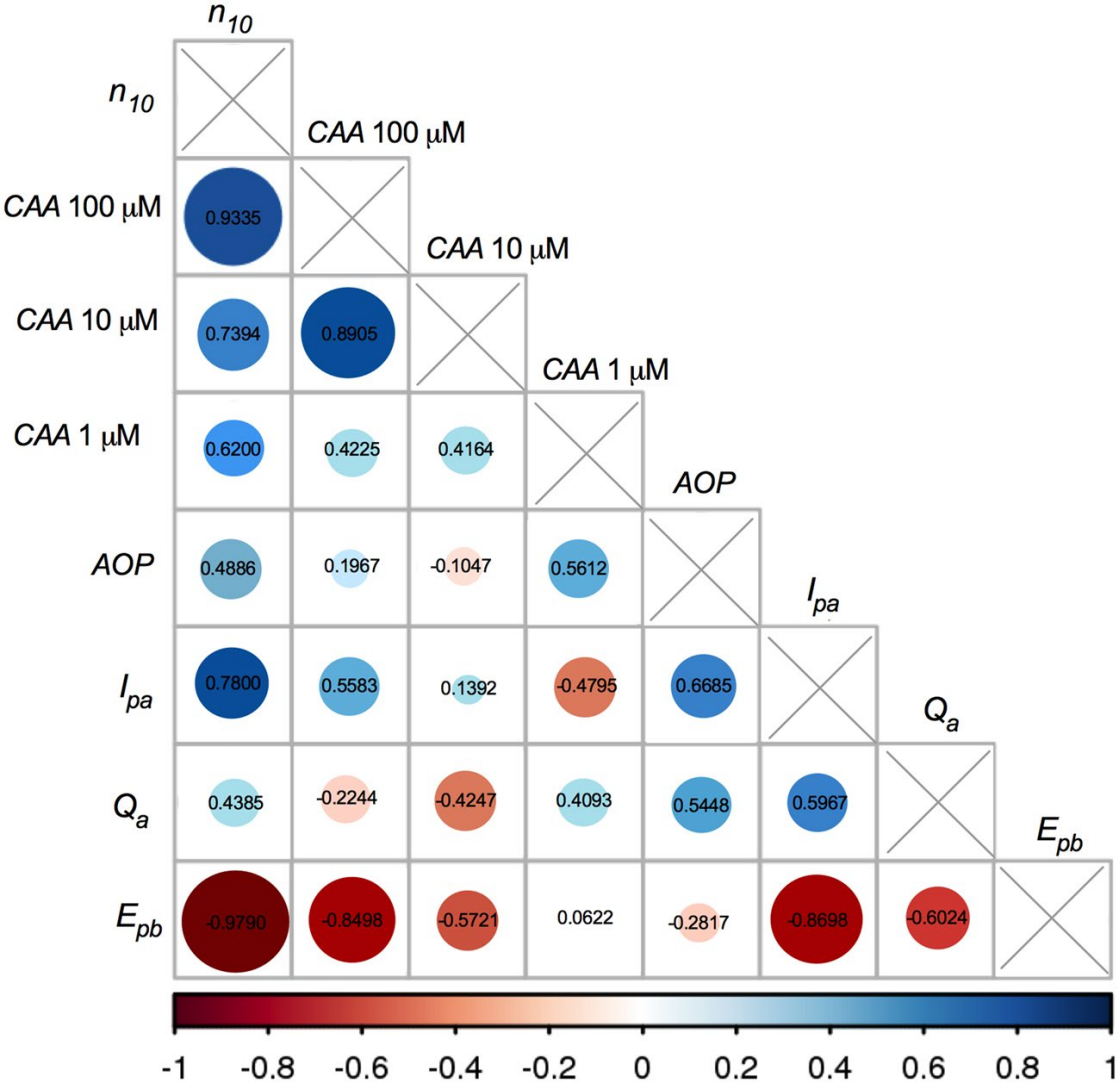
Correlation analysis between antioxidant activity parameters determined by DPPH test, PT and DPV as well as CAA test examined using Pearson’s coefficients. The size and colour of the circles represent the degree of correlation between the indicators; red is negative, and blue is positive. The corresponding value of Pearson’s coefficients is given on each circle.

The first series of experiments concentrated on chemical and electrochemical determinations of parameters characterizing oxidation of individual and 1:1 mixed flavonoids (representatives of flavonol and flavanone group), separately in an aglycone (Q, N−) and glycoside (R, N+) form. The correlations between the results of these measurements are given in Fig. 8. The initial assessments performed by the most popular DPPH batch test showed that although both flavanones (N−, N+) seemed very weak antioxidants, they increased the antioxidant activity of mixtures, both QN− and RN+, in a synergistic manner (Fig. 2A). DPV analysis provided deeper explanation of this effect and pointed to the decisive impact of the kinetics of the reaction. It turned out, that the studied flavanones easily release electrons according to the high value of thermodynamic parameter *E*_*p,a*_ (Fig. 2C). Still, the kinetics of this process was too slow to be observable by DPPH and PT tests. The flavonols, strong antioxidants in DPPH test and PT, exhibited in DPV the opposite properties—unfavourable thermodynamics of electron release, but high rate of the oxidation process. The comparison of structures of intermediate radicals suggests that flavonol semiquinone radical is more stable than flavanone phenoxyl radical owing to the several stabilising mechanisms (Fig. 1). It follows that the stabilization of the radical intermediate is of crucial importance for the kinetics of the oxidation reaction and, consequently, for the reductive activity of the compounds investigated.

Final antioxidant activity of the mixtures studied is magnified via the thermodynamic factors, favourably impacting oxidation process, which subsequently improved the reaction kinetics (Fig. 2B–F). A more detailed mechanism of this enhancement remains currently unexplained, most probably it involves specific flavonol/flavanone interactions. This enhancement was observed not only in a test tube, but also under cellular circumstances as demonstrated by CAA assay in which antioxidant activity of both mixtures, QN− and RN+, displayed antioxidant activity higher than that of individual components (Fig. 4). The chemical part of our investigations revealed the discrepancy in antioxidant activity between individual compounds and their mixtures. One can point to the importance of the kinetics of the oxidation reaction for the overall antioxidant activity and suggest that interactions between individual components influence the redox properties of the mixture. It must be correct at least in this case, when the presence of flavonols (Q, R) in the mixture increased the ability of flavanones (N−, N+) to donate electrons.

The subsequent part of the research concentrated on the comparison of redox-related biological properties exhibited by individual flavonoids (Q, R, N−, N+) and their mixtures (QN− and RN+). HT29 human colon cancer cells served as a recommended for nutritional studies model of human alimentary tract^53^, however in this study some data interpretations also refer to the neoplastic nature of this cell line. It has been generally accepted nowadays, that redox balance is vital for cell survival and function; thus, exposure to exogenous antioxidants as well as ROS may modulate many cellular processes. Upon reductive stress, insufficient ROS abundance may alter cell signalling via redox dependent pathways^46^. The excess of ROS, on the other hand, leads to oxidative stress and increased risk of oxidative damage to cellular components. The biological starting point of this study was the assessment of the impact of antioxidants, individual and in mixture, on cellular growth; the activity that is dependent on cellular redox homeostasis, since the proper concentration of ROS is key for the activation of signalling that triggers cell proliferation^54^. The results of MTT cell viability test revealed substantial differences between treatments. Pure compounds did not impact cell proliferation significantly compared to control non-treated cells; cell growth attained a constant level at the broad range of concentrations. In contrast, both mixtures (QN−, RN+) significantly stimulated cell proliferation. This latter effect may not necessarily be related solely to antioxidant properties of mixtures, because CAA assay results for Q were similar to those for QN− mixture. Nonetheless, the mixtures, but not individual components, apparently better supported HT29 cells to deal with the residual oxidative stress, e.g. by restoring the optimal redox status; the effect observed previously for such strong antioxidants as catechins^55^. Although, the synergistic increase of antioxidant activity observed for mixtures may be perceived as a beneficial effect, the fact that such combinations of antioxidants stimulate the growth of cancer cells is not desirable. The pros and cons of antioxidants and ROS in cancer have been a subject of debate for some time and led to the conclusion that antioxidants may promote cancer through complex mechanisms^56^, which is also seen here.

Another interesting finding was that the undesirable effects of treatments, such as pro-oxidative activity of N− revealed by CAA assay as well as genotoxicity of Q observed in comet assay were smoothed away for the mixtures. The latter assay revealed that DNA damage caused by the highest studied concentration (100 μM) of pure Q was decreased to control level for the equimolar concentration of the mixture QN− (Fig. 5) that may be linked to improved antioxidant activity of QN− observed in electrochemical tests. In the case of cellular antioxidant activity assessment, aglycones exhibited more clear-cut antioxidant (Q) or prooxidant (N−) properties than corresponding glycosides (Fig. 4). N− at physiological concentrations did not override the redox buffering capacity of normoxic HT29 cells, while at higher concentrations than those found in human plasma exhibited a concentration-dependent pro-oxidative effect that decreased with exposure time. Generally, aglycones declined their initial impact on cellular redox status over the time course (Fig. 4). Both mixtures displayed enhanced antioxidant activity, apparently not influenced by pro-oxidative effect seen for individual compounds.

The differentiated impact of mixtures compared to individual components was also seen in the epigenetic version of the comet assay employed to monitor changes in DNA methylation. Individual flavonoids showed the tendency to decrease global DNA methylation in a concentration-dependent manner, with the exception of R, which did not influence this epigenetic modification (Fig. 6). Both mixtures also reduced global DNA methylation level, but no correlation with the concentration was noticed. It is worthy of note, that not only redox status may play a role upon combining polyphenols, since in the case of DNA methylation, the impact of studied flavonoids did not seem to be associated with their reducing properties. Active demethylation is known to involve iterative oxidation of methyl group in 5-methylcytosine to carboxy form^57,58^, thus antioxidants would be expected to block this process. In our experiments, we observed the opposite impact. Therefore, here most probably another mechanism was involved, which is inhibition of DNA methyltransferase1 (DNMT1)—the enzyme that catalyses the transfer of methyl groups to dinucleotide CpG structures in DNA. The blockade of methylation pattern maintenance leads to the passive demethylation over consecutive rounds of DNA replication. Our results are in line with other studies that demonstrated DNMT1 inhibition by quercetin^59^. Also some flavanones, including N− were shown to inhibit DNMT1 activity in nuclear extracts of human oesophageal squamous cell carcinoma KYSE-510 cells^60^. Our study showed also that mixing Q with N− caused a remarkable drop in DNA methylation level at all tested concentrations of this mixture. A similar outcome was observed for RN+; however, the hypomethylation of DNA occurred to a lesser extent. All these observations may be of interest from a therapeutic point of view. DNA methylation pattern in cancer is characterised on one hand by global loss of methylation at gene bodies and intergenic regions leading to attenuation of the genome stability^61^, on the other hand, by hypermethylation of CpG-rich regions in promoters and transcriptional silencing of expression of tumour suppressor genes (TSGs)^61^. Thus, DNA hypomethylation induced by polyphenols may restore the expression of silenced TSGs genes and also gradually increase cancer genome instability to the point leading to cell death.

The described cellular effects of polyphenols studied showed that not only content or composition or bioavailability, but also interactions between components modulate electrochemical as well as biological properties of mixtures. This conclusion was also vividly supported by the last activity analysed in this study, i.e., modulation of expression of a wide spectrum of genes associated with the antioxidant defence and oxidative stress response. The Venn diagram (Fig. 7C) summarises the impact of investigated flavonoid aglycones on the modulation of gene expression. In the case of pure compounds (Q, N−) at 1 μM, only one gene (*GTFZI*) was found to be affected by both flavonoids. However, N− caused down-regulation of this gene, while Q increased its expression. This impact was not maintained at higher concentration of pure compounds nor was seen for their mixture at any dose. N− and the mixture QN− down-regulated also two other genes, *CCL5* and *MT3*, at 1 μM and three genes embracing *CYGB*, *MT3* and *MSRA* at 10 μM. Most surprisingly, no similarities in regulation of expression of genes were found between Q and QN− nor between Q, N− and QN− at any of the investigated concentrations. Moreover, the mixture QN− changed significantly the expression of three other genes (*APOE* and SEPP1 down-regulation, *PRDX5* up-regulation) whose transcriptional activity was not affected by any of the individual components.

In conclusion, our study demonstrates that biological properties of polyphenol mixtures are not just the combination of enhanced or weakened activities exhibited by individual components. These observations indicate that bioactivity of phytochemicals in mixtures must be a result of interactions between components leading to the emergence of a new substance with novel chemical and biological properties that are difficult to predict. The results of determinations carried out in our study do not merely support, but actually, broaden the idea of food synergy concept emphasizing the fact that even minor modifications in the composition of a mixture of foodborne phytochemicals (probably also food ingredients of other origins) create a new entity whose impact on human health may not necessarily resemble that of individual components. This notion undermines the current way dietary supplements are designed, which build on health claims established from research on isolated compounds. From a dietary chemoprevention perspective, the presented study explains why the current approach emphasizing the use of isolated bioactive food components was unable to match the epidemiological observations made for the whole foods people ingest. If food supplements are to offer true long-term health benefits to individuals, it is vital that combinations of putative agents are studied together and within a biological context.

## Materials and methods

### Chemicals, reagents

The following bioactive compounds were used for the study: quercetin (Q), rutin (R), naringin (N+) and naringenin (N−) from Sigma-Aldrich (USA). Analytical grade ethanol and methanol from POCH (Poland) as well as DMSO from Sigma-Aldrich (USA) were used. QPLUS185 system from Millipore (USA) was used to purify water. For antioxidant activity assessments by spectrophotometric test, 1-diphenyl-2-picrylhydrazyl (DPPH) from Sigma-Aldrich (USA) was applied. 0.1 M sodium phosphate buffer prepared by dissolving Na_2_HPO_4_∙12H_2_O and NaH_2_PO_4_∙2H_2_O (Sigma-Aldrich, USA) in deionized water was used in electrochemical studies. The working electrode and the electrochemical cell were cleaned with the solution of 10 mM potassium permanganate (Sigma-Aldrich, USA) in 95% H_2_SO_4_ (v/v) (POCH, Poland). The reference electrode was stored in 3 M KCl (Sigma-Aldrich, USA) dissolved in deionized water. All reagents utilized in the cell culture (PBS, McCoy’s 5A medium, trypsin, foetal bovine serum, antibiotics) were purchased from Sigma-Aldrich (USA). PBS solution was prepared by dissolving one tablet in 200 mL purified water. Thiazolyl blue tetrazolium bromide (MTT) from Sigma-Aldrich (USA) was applied in MTT test. The OxiSelect Cellular Antioxidant Assay Kit was purchased from Cell Biolabs, Inc. (USA). The following reagents were used for comet assay: hydrochloric acid (HCl), low melting point agarose (LMP agarose), sodium chloride (NaCl), sodium hydroxide (NaOH), ethylenediaminetetraacetic acid (EDTA), 2-amino-2-(hydroxymethyl)-1,3-propanediol (Trizma-Base), Sybr Green I nucleic acid gel stain and Triton X-100 from Sigma-Aldrich (USA) as well as normal melting point agarose (NMP agarose) from Bioline (UK). Additionally, in methylation sensitive comet assay, proteinase K (Merck, USA), restriction enzymes (HpaII/MspI) and Tango buffer (Promega, UK) were applied. QIAshredder, RNeasy Mini Kit, RNase-Free DNase set, RT^2^ First Strand Kit, RT^2^ SybrGreen qPCR Mastermix, RT^2^ Profiler PCR Arrays for Oxidative Stress (PAHS 0065) from Qiagen (Germany) were used in genomic studies.

### Antioxidant activity by DPPH test

The determination of antioxidant activity of investigated antioxidants and their mixtures was carried out by spectrophotometric assay employing DPPH radical as described previously^13,55^. Firstly, the stock solution of DPPH radical was diluted with methanol until absorbance amounted to 0.9 ± 0.05 at 515 nm. Secondly, antioxidants and their mixtures were diluted appropriately with 70% ethanol to achieve concentrations falling within the linear range of the assay^13,55^. Then, the DPPH solution (1 mL) was mixed with the diluted samples (30 μL) and the absorbance was measured at 515 nm after 10 min at 37 °C. All reactions were carried out in 48-well plates. The absorbance measurements were performed with the aid of a TECAN Infinite M200 spectrophotometer (Tecan Group Ltd., Switzerland). The antioxidant activity of sample was recalculated to stoichiometry coefficient *n*_10_ as described previously, with modifications^55^. Briefly, the amount of radicals scavenged by the tested samples was calculated based on the Beer-Lambert law and the molar extinction coefficient of DPPH following the measurements performed after 10 min of reaction between the antioxidant(s) solution and radical^55^. The value of *n*_10_ is calculated as a tangent of the linear relationship between the number of µmoles of DPPH scavenged by 1 mL of antioxidant(s) solutions within a concentration range, where the “stock” solution has the concentration “100%” and the other concentrations are a fraction of 100% as defined by the dilution factors.

### Antioxidant activity by potentiometric titration and differential pulse voltammetry

Standard reduction potentials (*E*^0^) for Q and R were measured by potentiometric titration (PT) as described elsewhere^55^. In short, studied compounds and the titrant (K_3_[Fe(CN)_6_]) were dissolved in PBS. The concentration of purified compounds was 0.3 mg/mL. Mixtures contained 0.3 mg/mL of each compound. Measurements were carried out using JENCO 6230 N ORP-146C Micro Oxidation–Reduction equipment (USA) with the aid of Ag|AgCl reference electrode (RE) and a platinum measuring electrode. PTs were performed at 37 ± 0.01 °C that was maintained by Ultra Thermostat (PolyScience, USA). The equal volume of titrant was added to the analyte and steady potential was read. As a result, titration curves, *E* = f(*V*_*titr*_), were analysed with the aid of SigmaPlot Version 13.0 software (Systat Software Inc., UK) by fitting of the sigmoidal, 5-parameters mathematical model to experimental data^55^. Potential at equivalence point (*EP* vs. RE) was read directly from this model based on parameter *a*_*2*_. It is equal to the volume of the titrant added at the inflection point. Finally, the values of *EP* versus SHE (standard reduction potential, *E*^0^) were calculated. The correction term of the potential of the RE (ε) was established by titration of redox couples, FeCl_3_∙6H_2_O and Na_2_S_2_O3∙5H_2_O, characterized by known standard reduction potentials.

In turn, antioxidant power (*AOP*) of studied compounds and their mixtures was measured by differential pulse voltammetry (DPV) as shown before^13^ with modifications. Briefly, measurements were carried out using the Gamry Reference 600 potentiostat (Gamry Instruments, USA) containing a three-electrode system. Glassy carbon electrode (GC, 1.6 mm in diameter), platinum wire and Ag|AgCl electrode (Hydromet S.C., Poland) were applied as the working (WE), the auxilliary (AE) and the reference electrode (RE), respectively^13^. Before experiments, the surface of the WE was polished using alumina suspension (0.05 μm particles, Buehler, USA) on microcloth pads (MF-1040, BASi, USA) and then cleaned with distilled water and methanol. The studied compounds were diluted in DMSO and sodium phosphate buffer (pH = 7.4 ± 0.1), so as the final concentration of phosphate buffer was 0.1 M in sample. The buffer served as the supporting electrolyte. The concentration of purified compounds was 3 mM. Mixtures contained 3 mM of each compound. In order to eliminate the electrochemically reactive oxygen, the studied solutions were deoxidized by argon percolation before the measurements. DPV voltammograms for N−, N+, and mixtures: QN− and RN+ were recorded in the range − 0.2 to + 1.3 V, while for Q and R in the range − 0.2 to + 0.6 V vs. RE. The potential scan rate of 0.1 V·s^-1^, pulse height of 0.05 V and pulse time of 0.1 s at 25 ± 0.01 °C were set.

DPV voltammograms were analysed by SigmaPlot Version 13.0 software (Systat Software Inc., UK). The *AOP* values were calculated in two steps. Calculations considered not only anodic peak potential and current (*E*_*p,a*_; *I*_*p,a*_), but also the set potential and measured current at each point of the voltammetric curve. It allowed more precise values to be obtained than those reported in our previous work^13^. Firstly, parameter of antioxidant energy (*AOE*) was calculated according to Eq. 1:

1 $$AOE = - \frac{1}{{A_{WE} }}\mathop \sum \limits_{i}^{f} \left( {E + \varepsilon } \right) \cdot I \cdot dt\,[{\text{J}}\,{\text{cm}}^{ - 2} ]$$

where *A*_*WE*_ is the surface area of the WE (equal to 0.162 ± 0.004 cm^2^ in our study), *E* is a set potential versus RE [V], *ε* is a correctional factor taking into account the presence of the liquid junction between the WE and RE (here 0.103 V), *I* is the current measured versus the background current [A], *dt*—is the potential sampling time [*dt* = 0.5 s].

Secondly, *AOP* expressed in W cm^-2^ unit was calculated based on Eq. 2:

2 $$AOP = \frac{AOE}{{t_{f} - t_{i} }} \,[{\text{W}}\,{\text{cm}}^{ - 2} ]$$

where *t*_*f*_*-t*_*i*_—is the difference between the time of beginning of the oxidation peak (*t*_*f*_) and its end (*t*_*i*_).

In order to determine *A*_*WE*_, cyclic voltammetry for 1·10^–3^ M K_3_[Fe(CN)_6_] solution in 0.1 M KCl was performed. This value was calculated based on Randles-Ševčík equation^62^ from the slope of the anodic peak current as a function of square root of the scan rate, *I*_*p,a*_ = f(*v*^1/2^). The *ε* was measured in the same way as in potentiometric titration. Moreover, in the present work, the thermodynamic parameter of oxidation process was anodic peak potential corrected by liquid junction between WE and RE (*E*_*p,b*_ = *E*_*p,a*_ + *ε*), while kinetic parameters embraced charge transferred (*Q*_*a*_) and anodic current (*I*_*p,a*_).

### Cell culture

In the presented study, HT29 cell line (human colon adenocarcinoma) from the ATCC was used as a model of human intestine. The cells were maintained in McCoy's medium supplemented with antibiotics (100 U/mL streptomycin and 100 g/L penicillin) and foetal bovine serum (100 mL/L)^13^. The HT29 cell line was maintained at 37 °C under 5% CO_2_ atmosphere in a cell incubator (Heal Force)^13^. The cell line was employed between passages 6 and 11. Cultured cells were tested for mycoplasma contamination using Universal Mycoplasma Detection Kit from ATCC (USA).

### Cytotoxicity assessment

To determine the impact of purified antioxidants (Q, N+, R, N−) and their mixtures (QN−, RN+) on HT29 cells growth, MTT test was applied as described earlier^13,55^. Briefly, the exponentially growing cells were seeded in 96-well tissue culture plates (5 × 10^3^ cells per well in 0.18 mL of medium) and were left to settle for 24 h at 37 °C under 5% CO_2_. Then, the cells were treated for 6, 24 or 72 h with 0.02 mL of different concentrations of the pure antioxidants or their mixtures^13,55^. The antioxidants and their mixtures were dissolved in ethanol (naringenin, naringin and mixtures—30% (v/v), quercetin—40% (v/v), rutin—20% (v/v)). The final concentrations of purified compounds ranged from 10 nM to 100 μM. The mixtures contained equivalent concentrations of each compound (10 nM–100 μM). The final concentration of ethanol in culture media was 2% (v/v) in the case of rutin, 3% (v/v) naringenin, naringin and mixture as well as 4% (v/v) quercetin. After 6 and 24 h exposures, the medium was aspirated from the wells and replaced with 0.2 mL of fresh medium. The cells were incubated at 37 °C until 72 h of the total incubation time^13,55^. After 72 h of incubation, to all wells 0.05 mL of MTT solution (4 g/L) was added and the cells were maintained for further 4 h at 37 °C^13,55^. Then, medium was aspirated from wells and formazan crystals were dissolved in 0.05 mL of DMSO. The absorption of the obtained solutions was measured at 540 nm with the aid of TECAN Infinite M200 plate reader (Tecan Group Ltd., Switzerland)^13,55^. The treatments were performed as four technical replicates. Three independent repetitions of each treatment were performed. The impact of investigated samples on HT29 cells growth was expressed as percent of growth inhibition of cells exposed to individual antioxidants and their mixtures compared to control cells treated with the solvent only, whose growth was regarded as 100%^13,55^.

### CAA (cellular antioxidant activity) assay

CAA assay (The OxiSelect Cell Biolabs, Inc., USA) was used to evaluate the cellular antioxidant activity of compounds alone (Q, N+, R, N−) and in mixtures (QN−, RN+) in HT29 cells as described earlier^13,55^. The exponentially growing cells were seeded in 96-well tissue culture black plates with transparent bottoms for fluorescence measurements (3 × 10^4^ cells per well in 0.2 mL of medium) and were left to settle for 24 h at 37 °C under 5% CO_2_^13,55^. All antioxidants were dissolved in 10% ethanol. The cells were then treated with 500 times diluted solution of 2′,7′-dichlorofluorescin diacetate (0.05 mL) provided with the kit, and the same volume of different concentrations of antioxidant samples for 1, 3 or 6 h. The final concentrations of purified compounds ranged from 1 to 100 μM. The mixtures contained equivalent concentrations of each compound (1–100 μM). The control cells were treated with 10% ethanol only (v/v). The final concentration of ethanol in culture media was 5% (v/v). All treatments were carried out in three technical replicates and three independent experiments were performed. Subsequent steps were carried out according to the manufacturer’s recommendations (https://www.cellbiolabs.com). Calculations were performed as described earlier^13,55^.

### Genotoxic effects

To determine the genotoxic effects exhibited by individual antioxidants (Q, N+, R, N−) and their mixtures (QN−, RN+) in HT29 cells, comet assay procedure was applied as described previously^55^. The exponentially growing HT29 cells were seeded in 24-well tissue culture plates (10^5^ cells per well in 1.8 mL of medium) and were allowed to settle for 24 h at 37 °C under 5% CO_2_^55^. Then, the cells were treated for 24 h with 0.2 mL of different concentrations of the antioxidants alone or in mixtures. The final concentrations of purified compounds ranged from 1 to 100 μM. The mixtures contained equivalent concentrations of each compounds (1–100 μM). The final ethanol concentration in the culture medium was 3% (v/v). The cells used as negative controls were treated with solvent only. After treatment time, the medium was aspirated from the wells and the cells were washed with 0.5 mL PBS. The cells were then detached using 0.2 mL of trypsin solution (0.5 g/L)^55^. The activity of trypsin was halted by adding 1.8 mL of complete growth medium to each well. The cells were re-suspended, counted and aliquoted into 1.5 mL tubes (30 × 10^3^ cells per tube). The cell suspension was centrifuged (100 × g, 5 min, 4 °C). The cell pellets were washed with 1 mL of PBS and centrifuged again (100 × g, 5 min, 4 °C)^55^. Then, PBS was discarded and the cells were re-suspended in 150 μL of 0.5% LMP agarose in water pre-warmed to 42 °C and 40 μL of this mixture was placed as two spots on a microscope slide pre-coated with 1% normal melting point agarose (NMP agarose). The slides were covered with coverslips. The agarose was allowed to set by placing the microscope slides on an ice-cold tray for at least 5 min^55^. Three slides with two repetitions on each were prepared for every concentration of the tested substances. After overnight lysis in a high salt alkaline buffer (2.5 M NaCl, 0.1 M EDTA, 0.01 M Tris, 1% Triton X100, pH 10), the slides were accommodated into a Bio-Rad Sub-Cell GT electrophoresis platform (UK), covered with cold electrophoresis buffer (0.3 M NaOH, 1 mM EDTA, pH 13) and chromatin was allowed to unwind for 25 min before electrophoresis^55^. Electrophoresis was conducted at 26 V and 300 mA (0.75 V/cm) for 30 min in darkness at 4–8 °C. After this step, the slides were washed firstly using PBS and then water. Subsequently, the DNA was stained with SybrGreen in TE buffer (0.1 M Trizma-Base, 1 mM EDTA, pH 7.5) for 20 min^55^. After staining, the slides were washed with distilled water for 5 min. DNA “comets” were analysed under a fluorescence microscope (Zeiss ImagerZ2, USA) coupled with a computerized slide scanning system (Metafer4, Germany). Comet analysis involved counting 200 consecutive nuclei per sample^55^. Genotoxicity of analysed samples was expressed as the *%DNA in the comet tail*. Three independent replicates of each treatment were performed.

### Global DNA methylation

For determination of global methylation of DNA, a modified comet assay procedure was developed. Methylation sensitive comet assay was performed according to Wentzel’s procedure with significant modifications^63^. The cells were seeded in 6-well tissue culture plates (4 × 10^5^ cells per well in 3.6 mL of medium) and were allowed to settle for 24 h at 37 °C under 5% CO_2_. Then, the cells were treated with 0.4 mL of different concentrations of the tested antioxidants or their mixtures for 24 h at 37 °C. The final concentrations of individual compounds and solvent were the same as used for gentoxic effect assessment. After incubation time, the medium was aspirated from the wells and the cells were washed with 2 mL of PBS. The cells were detached using 0.4 mL of trypsin solution (0.5 g/L). Then, 3.6 mL of medium was added to each well. The cells were re-suspended, counted and aliquoted into 1.5 mL tubes (30 × 10^3^ cells per tube). The cell suspension was centrifuged (100 × g, 5 min, 4 °C). The cell pellets were washed with 1 mL of PBS and centrifuged again (100 × g, 5 min, 4 °C). PBS was discarded and the cell pellet was re-suspended in 100 μL of 1% LMP agarose in water pre-warmed to 45 °C. Then, 40 μL of this mixture was placed as two spots on a microscope slide pre-coated with 1% normal melting point agarose (NMP agarose)^55^. The slides were then covered with coverslips and left to set on an ice-cold tray for at lest 5 min to solidify agarose^55^. Each treatment embraced a set of 3 microscope slides. After overnight lysis in a high salt alkaline buffer (2.5 M NaCl, 0.1 M EDTA, 0.01 M Tris, 1% Triton X100, pH 10), the slides were washed twice with water^55^. Tightly packed chromatin was unwound by treating the nuclei with 1.5 mM proteinase K solution (0.2 mL per slide)^63^. The slides were covered with parafilm, placed into a plastic container lined with a damp tissue, left for 10 min at 37 °C, then washed with water^63^. In this way, nucleoids were prepared for the digestion with restriction endonucleases (HpaII and MspI). Both enzymes recognize the same restriction sequence (5′-CCGG-3′), but show different sensitivity towards methylated cytosine^63^. HpaII is presumed to digest only non-methylated sequences. MspI can cleave non-methylated sequences as well as fully methylated sequences. Thus, the relative levels of DNA methylation of CpG sequence are reflected as the difference between the global amount of DNA within the comet tail observed with MspI digestion and HpaII digested nucleoids^63,64^. To create appropriate conditions for enzymatic digestion, 0.2 mL of Tango buffer diluted with molecular grade water in ratio 1:9 (v/v) was applied onto each slide in the set. The slides were covered with parafilm, accommodated into a plastic container lined with a damp tissue and left for 10 min at 37 °C^63^. Then, the excess of the buffer was removed. Each of three slides in the set was treated differently. Onto the first (control) slide, only 0.15 mL of diluted Tango buffer was applied. The agarose embedded nuclei on the second slide were treated with 0.15 mL of HpaII enzyme (0.37 μunits), while onto third slide the same volume of MspI was added (0.26 μunits). The enzyme solutions were prepared using diluted Tango buffer. The slides were covered with a parafilm and accommodated into a damp plastic container. The enzymatic digestion was carried out for 45 min at 37 °C. After digestion, the slides were twice washed with water. Further steps of the comet assay, such as electrophoresis and DNA staining, were performed as described in the section “Global DNA methylation”. DNA “comets” were analysed under a fluorescence microscope (Zeiss ImagerZ2, USA) coupled with a computerized slide scanning system (Metafer4, Germany). Comet analysis was performed with the aid of Comet score software (USA) and involved counting of 100 nuclei per sample. The mean *%DNA in the comet tail* was a measure of DNA fragmentation. To calculate the global DNA methylation (%CpG methylation), the following equation was used: %CpG methylation = 100—HpaII/MspI × 100, where HpaII/MspI is the ratio of the DNA percentage in the comet tail of nucleoid digested with HpaII and the DNA percentage in the comet tail of nucleoid digested with MspI^63^. DNA damage artefacts were accounted for by subtracting the DNA percentage in tail of the control sample from the values obtained for the samples digested with restriction enzymes. Three independent repetitions of each experiment were performed.

### Microarray analysis

Genomic analysis has been performed as shown before^13,55^. HT29 cells were seeded in 24-well tissue culture plates (10^5^ cells per well in 1.8 mL of medium) and were allowed to settle for 24 h at 37** °C** under 5% CO_2_. Then, the cells were treated for 24 h with 0.2 mL of different concentrations of the antioxidants alone (Q, N-) or in mixture (QN-). The final concentrations of investigated compounds ranged from 1 to 10 μM. The mixture contained equivalent concentrations of each compound. The cells used as negative controls were treated with the solvent only. The final concentration of ethanol in culture media was 3% (v/v). Isolation of RNA, reverse transcription and real-time PCR of array consisting of 84 genes involved in antioxidant response as well as data analysis were performed as described earlier^13^. Three independent repetitions of each treatment of cells were carried out.

### Statistical analysis

All values are expressed as mean ± SD of three independent experiments unless stated otherwise. The statistical significance of determinations of antioxidant activity in a cell free system using DPV and DPPH assay as well as in cellular models obtained by CAA test were examined by unpaired Tukey’s test (*p* ≤ 0.05). The results of genotoxicity and global DNA methylation analysed by comet assays were examined by one-way ANOVA with Dunnett's post-hoc test. These statistical analyses were performed using Prism 4.0 software package (GraphPad Software, Inc., USA). The statistical significance of changes in gene expression between samples and controls was also evaluated by unpaired Student’s t-test for each gene of interest using GenGlobe Data Analysis Center (Qiagen, USA). The level of statistical significance was set at *p* ≤ 0.05.

## Supplementary Information

Supplementary Information.

## Abbreviations

AE: Auxilliary electrode
*AOE*: Specific antioxidant energy
*AOP*: Antioxidant power
CAA: Cellular antioxidant activity
DNMT1: DNA methyltransferase 1
DPPH test: Test employing 1-diphenyl-2-picrylhydrazyl radicals
DPV: Differential pulse voltammetry
E^0^: Standard reduction potential
EP: Equivalence point
*E*_*p,a*_: Oxidation (anodic) peak potential versus reference electrode
*E*_*p,b*_: Peak potential versus standard hydrogen electrode
*I*_*p,a*_: Anodic current
q: Charge density of the process
N−: Naringenin
N+: Naringin
PT: Potentiometric titration
R: Rutin
RE: Reference electrode
Q: Quercetin
*Q*_*a*_: Charge transfer
WE: Working electrode
