# Supplementary material for: Interactions between polyphenolic antioxidants quercetin and naringenin dictate the distinctive redox-related chemical and biological behaviour of their mixtures
Source: Sci Rep. 2021 Jun 10;11:12282. doi: 10.1038/s41598-021-89314-0 (PMC8192515; doi:10.1038/s41598-021-89314-0)
Supplement: Supplementary file 1 — Supplementary Information. [file 41598_2021_89314_MOESM1_ESM.pdf]

Interactions between polyphenolic antioxidants quercetin and naringenin dictate the distinctive redox-related chemical and biological behaviour of their mixtures.

Monika Baranowska, Zuzanna Koziara, Klaudia Suliborska, Wojciech Chrzanowski, Michael Wormstone, Jacek Namieśnik, Agnieszka Bartoszek

Table S1. Genes involved in oxidative stress response and antioxidant defence system of cells included in the microarray

Symbol	Description	Class
**ALB**	Albumin	a
**ALOX12**	Arachidonate 12-lipoxygenase	c, d
**AOX1**	Aldehyde oxidase 1	d
**APOE**	Apolipoprotein E	a
**ATOX1**	ATX1 antioxidant protein 1 homolog (yeast)	a
**BNIP3**	BCL2/adenovirus E1B 19kDa interacting protein 3	f
**CAT**	Catalase	c
**CCL5**	Chemokine (C-C motif) ligand 5	e
**CCS**	Copper chaperone for superoxide dismutase	d
**CYBB**	Cytochrome b-245, beta polypeptide	d
**CYGB**	Cytoglobin	d
**DHCR24**	24-dehydrocholesterol reductase	c, f
**DUOX1**	Dual oxidase 1	b, c, d
**DUOX2**	Dual oxidase 2	b, c, d
**DUSP1**	Dual specificity phosphatase 1	a, g
**EPHX2**	Epoxide hydrolase 2, cytoplasmic	c
**EPX**	Eosinophil peroxidase	b
**FOXM1**	Forkhead box M1	g
**FTH1**	Ferritin, heavy polypeptide 1	h
**GCLC**	Glutamate-cysteine ligase, catalytic subunit	h
**GCLM**	Glutamate-cysteine ligase, modifier subunit	h
**GPX1**	Glutathione peroxidase 1	b, c
**GPX2**	Glutathione peroxidase 2 (gastrointestinal)	b, c
**GPX3**	Glutathione peroxidase 3 (plasma)	b, c
**GPX4**	Glutathione peroxidase 4 (phospholipid hydroperoxidase)	b, c
**GPX5**	Glutathione peroxidase 5 (epididymal androgen-related protein)	b, c
**GPX6**	Glutathione peroxidase 6 (olfactory)	b, c
**GPX7**	Glutathione peroxidase 7	b, c
**GSR**	Glutathione reductase	b, c
**GSS**	Glutathione synthetase	a
**GSTP1**	Glutathione S-transferase pi 1	b, c
**GSTZ1**	Glutathione transferase zeta 1	b, c
**GTF2I**	General transcription factor IIi	d
**HMOX1**	Heme oxygenase (decycling) 1	h
**HSPA1A**	Heat shock 70kDa protein 1A	h
**KRT1**	Keratin 1	h
**LPO**	Lactoperoxidase	b, c
**MB**	Myoglobin	a
**MBL2**	Mannose-binding lectin (protein C) 2, soluble	e
**MGST3**	Microsomal glutathione S-transferase 3	b
**MPO**	Myeloperoxidase	b, c
**MPV17**	MpV17 mitochondrial inner membrane protein	d
**MSRA**	Methionine sulfoxide reductase A	c
**MT3**	Metallothionein 3	a
**NCF1**	Neutrophil cytosolic factor 1	d
**NCF2**	Neutrophil cytosolic factor 2	d
**NOS2**	Nitric oxide synthase 2, inducible	c
**NOX4**	NADPH oxidase 4	d
**NOX5**	NADPH oxidase 5	d

**NQO1**	NAD(P)H dehydrogenase, quinone 1	c
**NUDT1**	Nudix (nucleoside diphosphate linked moiety X)-type motif 1	a
**OXR1**	Oxidation resistance 1	a
**OXSR1**	Oxidative-stress responsive 1	a
**PDLIM1**	PDZ and LIM domain 1	a
**PNKP**	Polynucleotide kinase 3'-phosphatase	a
**PRDX1**	Peroxiredoxin 1	b, c
**PRDX2**	Peroxiredoxin 2	b, c
**PRDX3**	Peroxiredoxin 3	b, c
**PRDX4**	Peroxiredoxin 4	b, c
**PRDX5**	Peroxiredoxin 5	b, c
**PRDX6**	Peroxiredoxin 6	b, c
**PREX1**	Phosphatidylinositol-3,4,5-trisphosphate-dependent Rac exchange factor 1	d
**PRNP**	Prion protein	a
**PTGS1**	Prostaglandin-endoperoxide synthase 1	b
**PTGS2**	Prostaglandin-endoperoxide synthase 2	b
**PXDN**	Peroxidasin homolog (Drosophila)	b
**RNF7**	Ring finger protein 7	a
**SCARA3**	Scavenger receptor class A, member 3	a
**VIMP**	Selenoprotein S	e
**SEPP1**	Selenoprotein P, plasma, 1	a
**SFTPD**	Surfactant protein D	d, e
**SIRT2**	Sirtuin 2	a, f
**SOD1**	Superoxide dismutase 1, soluble	c, d
**SOD2**	Superoxide dismutase 2, mitochondrial	c, d
**SOD3**	Superoxide dismutase 3, extracellular	c, d
**SQSTM1**	Sequestosome 1	e, f, g
**SRXN1**	Sulfiredoxin 1	a, c
**STK25**	Serine/threonine kinase 25	a
**TPO**	Thyroid peroxidase	b, c
**TTN**	Titin	b, c
**TXN**	Thioredoxin	a
**TXNRD1**	Thioredoxin reductase 1	c
**TXNRD2**	Thioredoxin reductase 2	c

a - antioxidant activity; b - peroxidase activity; c - oxidoreductase acivity; d - involved in superoxide release and metabolism; e - relevant for inflamation; f - apoptosis inducers; g- relevant for cell proliferation; h - other involved in oxidative stress response

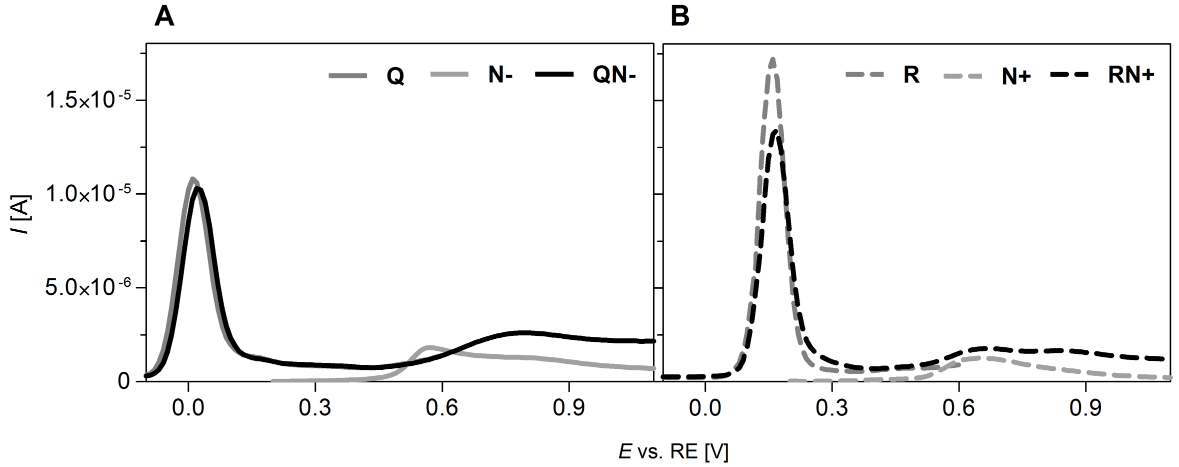

Fig. S1. The comparison of voltammetric curves obtained for individual flavonols, flavanones and their mixtures with the aid of DPV method.
